# Research with and about user participation: potentials and challenges

**DOI:** 10.1007/s40520-017-0750-7

**Published:** 2017-03-30

**Authors:** Marianne Kylberg, Maria Haak, Susanne Iwarsson

**Affiliations:** Department of Health Sciences, Faculty of Medicine, Lund University, Box 157, 221 00 Lund, Sweden

**Keywords:** User involvement, Transdisciplinary research, Participatory design

## Introduction

The need of a comprehensive public health approach that responds to the needs of the aging population has been stated as one way to handle the world-wide ongoing demographic changes. One crucial issue is to whom we leave to describe the needs of older people and decide on priorities in research and societal development. Research involving users is gaining interest, in medicine and health sciences, as well as in other fields of inquiry. Such approaches are seen as positive, not the least among politicians and research sponsors. As yet, however, the evidence base for the effects and outcomes of research with user participation is insufficient and scattered.

User participation in research concerns involvement that goes beyond just providing, for example, blood samples, answering surveys, and being tested or interviewed. Instead of being a research subject, the user is an active partner in the research process or in the development of evidence based activities in healthcare [1]. A broad definition of “users” includes all parties that are interested in and/or beneficiaries of research. This means that users are senior citizens in the general aging population and vulnerable people with specific characteristics and needs. Users are also informal carers, health care, social services, and industry professionals, as well as public agency, policy-maker, and interest organization representatives. To capture the diversity of the population, it is important to also pay attention to gender, age, ethnicity, socio-economic factors and physical, and psychological and intellectual functional ability.

The purpose of involving users in research has its origin in ideas relating to empowerment; to support individuals to take control over their own situation, involving a striving to shift power in the research process from the researchers to those the research concerns. The research process is then characterised by being carried out with the users as partners, with continuous feedback for mutual reflection and action between the parties as an important principle. The fundamental view of knowledge is, therefore, important to consider, not the least in the medicine and health sciences context where scientific knowledge is generally assigned higher value than tried and tested experience. With user participation in research, this view of knowledge is problematized and questioned. Users are seen as representatives of various groups and as experts on their situations and conditions [2]. It is necessary, therefore, to take into consideration the experience and wishes of patients and relatives as well as the relevance and benefit to the field of various activities. Researchers might be provoked by such recommendations, but need to expand their view of knowledge and take more interest in the contributions that users are able to make to research.

## Potentials and challenges of user participation

The positive effects of user participation in research are often taken for granted, but alongside potentials, there are many challenges involved (Table 1). User participation is expected to generate better understanding among researchers of relevant and urgent fields of research [1]. However, the change of role involved in being an active partner in research may represent a challenge for users and is thus important for the researcher to be responsive to. Another challenge lies in conducting the dialogue on equal terms and converting the users’ expressed problem areas or ideas into issues that can be scientifically studied [3].

**Table 1:** Potentials and challenges of research with and about user participation

	Potentials	Challenges
Fields of research/issues	The researcher is helped to identify and prioritise relevant research issues. The researcher gets a better understanding of relevant and urgent fields of research	Scientific and ethical conflicts may arise on account of differences in understanding and expertise between researcher and users
Methodology	As a data collector, a user can contribute to openness of the people interviewed and pave the way for deeper dialogue, producing richer or deeper data	Difficulties balancing scientific requirements such as reliability against participant’s subjective experiences
Execution	Positive effects—appreciated, noticed and increased self-confidence—for users involved in research that enable them to cope with the situation as a patient	Researcher and users may make different interpretations of collected data based on their knowledge and understanding of what is being studied. It is often time-consuming

As in all research, the recruitment of participants for active involvement in research is important. Most important, users should be able to represent a broader perspective than his/her own. With the intention to make contact with and include participants from specific contexts and with a variety of backgrounds, it is common to approach interest groups. This entails a risk of only reaching users who are much too focused on their personal situation and, therefore, unable to relate their own needs to the needs of others [4]. Thus, the recruitment process requires careful consideration.

Circumstances that can make it difficult to involve users in the research process include poor health, the time involved, and the challenges of travelling from home to various activities. An example of ethical aspects to take into account is the extent to which the experiences expressed may be used and reported, and in what format. There is a risk of users feeling that their statements are not taken seriously or the results published do not reflect their views. The time required for participation is particularly important to consider in relation to the users’ expectations about their ability to influence and change their own situation and that of others [5].

Involving users in data collection has both positive and negative impacts, both for those involved and for the results. In the role as data collector, a user can contribute to greater openness in terms of the experiences of the people interviewed and pave the way for a deeper dialogue, producing richer or deeper level data [2, 4]. There are challenges as well, not least of an ethical nature, when people with similar experiences meet in the roles of interviewer and interviewee in a research project. Moreover, it is important to consider how the material collected may be influenced by the person who collects it [4]. Researchers and users do not usually share experiences and understanding of what it is like to live in a certain situation, which may create a distance between them. Researchers and users may also make different interpretations based on their own knowledge and understanding of what is being studied. Summing up on potentials and challenges, ideally, user participation entails active involvement throughout the research process, which places demands on all parties [1, 3, 4] that should not be underestimated.

To evaluate the effects of user participation in research is complex and challenging. The evaluation initiatives hitherto reported are usually based on anecdotal evidence which indicates that this is a research area in need of development. A literature overview by Brett et al. [5] showed that in a large number of published papers, the effects of research with user participation were reported in different ways, with effects on three levels: for the individual users, for the target groups affected, and for the researchers. The positive effects for individual users are that they are appreciated and noticed, their self-confidence is increased, and they are able to cope with the situation as patient better. Target groups become more aware of their situation, and their knowledge about their specific problems is enhanced. However, users express that they would like more preparation and training for their tasks. Making conscious use of user participation in research, the researchers develop increased understanding of their own research field, acquire more respect for the users, and form stronger contacts with the groups of users with whom they conduct the research. At the same time, challenges associated with user participation in the research process are described, not least as extra time is required (Table 1). Several authors pointed out the increasingly common requirements for user participation from research sponsors, which mean that funds for user participation must be set aside in the project budget.

## Research needs to increase the knowledge base for user participation

As stated in our definition of users, not only users in terms of patients, clients, or other private individuals are involved in research. Representatives of trade associations, professional groups, public authorities, and political bodies also participate as users in research. There are many initiatives driving the development of knowledge about research with and about user participation forwards, but the scientific knowledge base is weak. The degree and importance of user participation are illustrated and discussed in various ways in the literature.

The published studies target many different user groups and represent a wide range of disciplines such as medicine and health sciences, gerontology, media and communication science, architecture, industrial design, computer science, and social work. Most of the studies have been carried out on a small scale within a specific activity in a national context, but there are examples of cross-national studies. The majority are qualitative studies or literature overviews [5]. Qualitative research is valuable for developing in-depth knowledge, but there is a risk that the build-up of knowledge will be a slow process. Therefore, it is necessary to develop a broader arsenal of methods that lays the foundations for the development of generalisable knowledge and cumulative knowledge building. There is also a significant need for more reliable, valid methodology for evaluation of research with user participation.

In conclusion, there is a growing volume of literature on research with and about user participation. Conceptual definitions and theory are insufficiently developed, and similar terms are used to describe both research with user participation and research about user participation without any distinction. In many cases, the fundamental approaches relating to the theory of the science require clarification. While the research field is hard to overview and analyse the recommendations and conclusions presented in varied, contexts have much in common, indicating that the range of knowledge is in the process of being consolidated. For researchers aiming to improve health and well-being for the aging population knowledge of the needs of older people are crucial. Since research sponsors often ask for user participation in their projects, it is important to enhance the knowledge about the challenges of involving users in research and how to handle them best, ultimately creating favourable conditions for future research aiming to create opportunities to handling the demographics changes.
